# Psychological resilience and quality of life among middle-aged and older adults hospitalized with chronic diseases: multiple mediating effects through sleep quality and depression

**DOI:** 10.1186/s12877-023-04473-1

**Published:** 2023-11-17

**Authors:** Jiashuang Xu, Lin Zhang, Hong Sun, Ziyun Gao, Meiding Wang, Mengya Hu, Qiqi Ji, Leilei Guo

**Affiliations:** 1 School of Nursing, Jinzhou Medical University, No.40, Section 3, Songpo Road, Linghe District, Jinzhou City, Liaoning Province People’s Republic of China; 2 Department of Internal Medicine Nursing, School of Nursing, Wannan Medical College, Higher Education Park, 22 Wenchang West Road, Wuhu City, An Hui Province People’s Republic of China

**Keywords:** Middle-aged and older adults, Chronic diseases, Psychological resilience, Sleep quality, Depression, Quality of life

## Abstract

**Background:**

The present study is intended to examine the multiple mediating roles of sleep quality and depression in the relationship between psychological resilience and quality of life in middle-aged and older adults hospitalized with chronic diseases.

**Methods:**

From October 2, 2021, to February 27, 2022, a questionnaire survey was conducted using a multistage stratified sampling method among 339 middle-aged and older adults (45 years and over) hospitalized with chronic diseases. These participants were recruited from a hospital in Zhejiang Province, China. The questionnaire included the Aged Cumulative Disease Rating Scale, the Psychological Resilience Scale, the Pittsburgh Sleep Quality Index Scale, the Depression Scale, and the Quality-of-Life Scale. A descriptive analysis was performed to characterize the sample. Linear regression was utilized to evaluate the relationship between psychological resilience and quality of life. Amos 24.0 was used to analyze the multiple mediated effects of sleep quality and depression.

**Results:**

Psychological resilience exerted a remarkable direct effect on the quality of life in middle-aged and older adults hospitalized with chronic diseases (β = 0.239, 95% CI = 0.125–0.354), which represented 52.98% of the total effect. Through three significantly mediated pathways indirectly affect the quality of life: (1) through the sleep quality pathway (β = 0.115, 95% CI = 0.056–0.201), which represented 25.39% of the total effect; (2) through the depression pathway (β = 0. 060, 95% CI = 0.023–0.114), which represented 13.24% of the total effect; and (3) through both the sleep quality and depression pathway (β = 0. 038, 95% CI = 0.019–0.074), which represented 8.39% of the total effect. The total mediating effect was 47.02%.

**Conclusions:**

Sleep quality and depression mediate the relationship between psychological resilience and quality of life in middle-aged and older adults hospitalized with chronic diseases. Therefore, healthcare professionals and stakeholders should be concerned about the sleep status and mental health of middle-aged and older adults hospitalized with chronic diseases, strengthen their attention to psychological resilience, and provide interventions and treatment measures for hospitalized patients who have sleep problems and depressive tendencies.

## Background

Chronic non-communicable diseases (often simply referred to as "chronic diseases") encompass conditions like chronic respiratory diseases, cardiovascular and cerebrovascular diseases, and diabetes mellitus. These diseases are characterized by a stealthy onset, prolonged course, and resistance to cure [1]. As China experiences an accelerated aging population, the prevalence of chronic diseases among middle-aged and older adults is rising annually [2]. Current statistics indicate that China has approximately 300 million chronic disease patients. Notably, more than half of these patients are middle-aged and older adults (≥ 45 years old), and a significant majority require frequent hospitalizations [3–5]. During their hospital stays, these patients typically undergo numerous medical examinations and treatments. Such interventions, while necessary, often result in pain, discomfort, and other physical side effects, thereby impacting their overall quality of life [6]. The concept of "quality of life" reflects an individual's perception of their life conditions within specific cultural and value contexts. It intertwines with one's aspirations, expectations, standards, and concerns, emphasizing personal values, self-realization, and societal responsibilities [7]. Moreover, quality of life serves as a predictor for hospitalization rates, mortality, and survival duration. It is also a paramount indicator in evaluating both medical outcomes for patients and the quality of healthcare provided [8]. Given these considerations, assessing and enhancing the quality of life for middle-aged and older adults hospitalized with chronic diseases becomes a matter of utmost importance.

The quality of life of middle-aged and older adults hospitalized with chronic diseases is influenced by a variety of factors, among which psychological resilience is particularly critical [9]. Psychological resilience is the ability of individuals to adapt, adjust, and recover in the face of adversity, stress, and challenge [10]. Numerous findings have shown that psychological resilience has a variety of positive effects on hospitalized patients with chronic diseases, including improved ability to perform daily activities, reduced adverse outcomes due to somatic dysfunction, increased well-being, and life satisfaction, reduced risk of death, as well as improved quality of life [11, 12]. Priya Sehgal et al.'s study on the connection between psychological resilience and quality of life revealed a positive correlation [13]. Ovidiu Popa-Velea et al. verified that psychological resilience has a preserving role in the quality of life of middle-aged and older adults hospitalized with chronic diseases [14]. Therefore, exploring psychological resilience can enhance the quality of life of middle-aged and older adults hospitalized with chronic diseases.

Sleep quality plays an essential role in maintaining the quality of life in middle-aged and older adults hospitalized with chronic diseases. Past research has revealed that chronic disease symptoms and unfamiliar hospital environments often lead to impaired sleep in hospitalized patients [15]. Low-quality sleep not only leads to patient's fatigue, poor concentration, and depressed mood during the day, but also leads to motor function decline and cognitive decline, increases the risk of obesity, dementia, and cardiovascular and cerebrovascular complications, and ultimately has a serious negative impact on their quality of life [16–18]. In addition, a study on hospitalized patients with rectal cancer showed a negative relationship between psychological resilience and sleep quality [19]. Another study of SLE hospitalized patients found that psychological resilience had a protective effect on sleep quality. Psychological resilience often reduces psychological stress-induced sleep disturbances and helps patients adopt more positive coping styles to reduce the incidence of sleep problems [20]. These results imply that sleep quality is closely related to psychological resilience and quality of life in middle-aged and older adults hospitalized with chronic diseases. However, there has been a relative absence of investigation into whether sleep quality mediates the relationship between psychological resilience and quality of life.

Middle-aged and older adults hospitalized with chronic diseases often suffer from sleep and psychological problems [21]. Depression, as a common psychological problem, is an important indicator for evaluating mental health [22]. It was found that the prevalence of depressive symptoms among hospitalized middle-aged and older adults with chronic diseases is increasing every year [23]. Depression is a category of mood disorders primarily characterized by significant and persistent mood depression [24]. Ki Jung Chang et al. showed that depression and anxiety hurt sleep quality in hospitalized patients [25]. Hong Fang et al. found that sleep disturbance is a key symptom of depression and that sleep disturbance may exacerbate other clinical symptoms in hospitalized patients such as fatigue, lethargy, irritability, high risk of suicide, cognitive impairment, and decreased quality of life [26–28].

Additionally, with the advancement of positive psychology research, scholars have explored the relationship between psychological resilience and depression [29]. Currently, it has been indicated that psychological resilience is a critical predictor of the onset and severity of depression in hospitalized patients with chronic diseases [30, 31]. Levels of psychological resilience were found to be strongly adversely correlated with anxiety and depression in a study of breast cancer hospitalized patients [32]. Another study on heart failure hospitalized patients reached a similar conclusion, finding that psychological resilience can reduce the impact of stress and trauma on depression and act as a buffer against the negative effects of depression [33]. In addition, Saba Moussavi et al.'s study found that depression not only affects the process of recovery of hospitalized patients but also further reduces their physiological functioning, which ultimately has a serious impact on their overall quality of life [34, 35]. Considering the argument above, depression might act as a mediator between psychological resilience and quality of life.

**Fig. 1:**
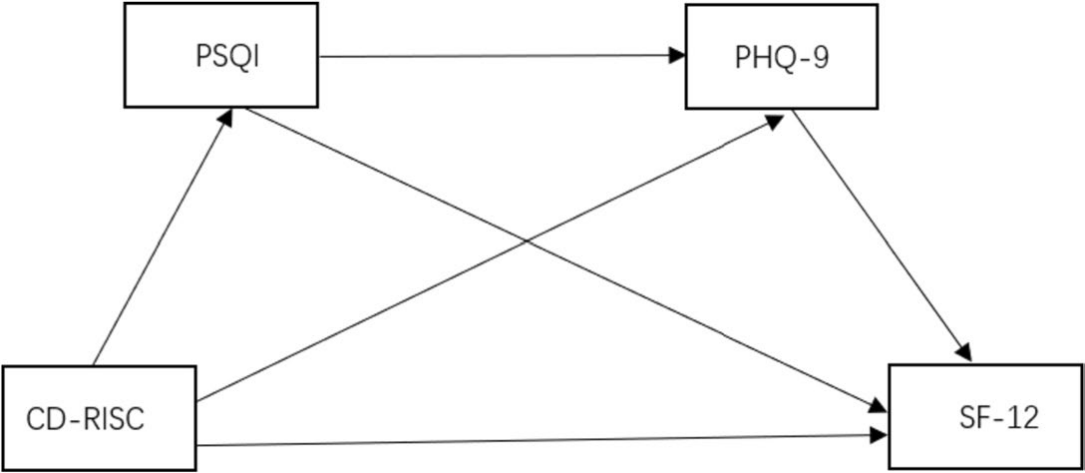
Hypothesized model

According to the above analysis, sleep quality and depression may play multiple mediating roles between psychological resilience and quality of life in middle-aged and older adults hospitalized with chronic diseases. Hence, three hypotheses were put forth to build the study's hypothetical model (Fig. 1). First, it was hypothesized (H1) that psychological resilience could influence the quality of life through sleep quality. Second, it was hypothesized(H2) that depression acted as a moderator between psychological resiliency and quality of life. Third, it was hypothesized (H3) that depression and sleep quality could act as chain mediators between psychological resilience and quality of life.

## Methods

### Participants

A multistage stratified sampling method was adopted for data collection, which was implemented from October 2021 to February 2022 in Shang Cheng District, Hangzhou City, Zhejiang Province, China. Calculations are made using a table of random digits. First, a third-class hospital in Shang Cheng District was chosen at random. Second, various hospital departments were chosen at random. Thirdly, randomly selected middle-aged and older adults hospitalized with chronic diseases (≥ 45 years) from the survey stations established in each section. The specific types of chronic diseases included are detailed in Table 2. Finally, 355 middle-aged and older adults hospitalized with chronic diseases who met the study's inclusion criteria were identified and approached for face-to-face surveys in a dedicated survey room. Questionnaires were delivered in total 355 and 339 valid questionnaires were recovered, with a valid response rate of 95.49%. The subjects were chosen based on the following inclusion criteria: (i) permission to participate [36]; (ii) clear consciousness and vocal communication without obstructions; (iii) No documented history or diagnosis of neurological or psychiatric diseases; and (iv) age 45 or older.

Before the survey, seventeen investigators received standardized training to improve their communication skills and understanding of the scoring methodology. Patients with chronic diseases were provided with questionnaires and asked one-on-one questions by researchers after giving their informed consent; patients then filled out the surveys. The declaration of Helsinki is followed by the application of all methods.

### Aged cumulative disease rating scale

The scale was created by William L. Leidy et al. in 1980 and was used to evaluate the prevalence and rate of the severity of disease in specific organ systems of individuals [37]. A total of 13 entries were included, with a scale ranging from 0–4 (0 being none, 1 being less severe, 2 being severe, 3 being more severe, and 4 being extremely severe). Higher scores represent co-morbidity at higher levels. In this study, the scale was used only to determine disease conditions of specific organ systems in middle-aged and older adults to better understand their health status and disease-related influences.

### Psychological Resilience Scale (Conner-Davidson Resilience Scale, CD-RISC)

The scale was created by American psychologists Connor and Davidson in 2003 based on the Posttraumatic Stress Disorder Research Project [38]. It was frequently used to gauge both the general population's and clinical patients' levels of psychological resilience. The scale included five factors covering personal ability, tolerance of negative feelings, positive acceptance of change, power of control, and spiritual influence, for a total of 25 items. A 5-point scale (0 is never like this, 1 is rarely like this, 2 is sometimes like this, 3 is often like this, 4 is always like this) was adopted, with scores ranging from 0 to 100, the higher the score, the higher the psychological resilience. The Cronbach's alpha coefficient for this scale was 0.933, and the KMO measure was 0.926.

### Pittsburgh Sleep Quality Index scale (PSQI)

The scale was created by Buysse et al. in 1989 to assess the quality of sleep of subjects during the most recent month [39]. It was composed of seven ingredients: A sleep quality, B sleep onset time, C sleep duration, D sleep efficiency, E sleep disorder, F hypnotic drugs, and G daytime dysfunction. Each item was given a value between 0 and 3, and the sum of those ratings was used to calculate the overall PSQI score, which varied from 0 to 21. Higher scores indicated less sleep quality. The Cronbach's alpha coefficient for this scale was 0.772, and the KMO measure was 0.783.

### Depression scale (PHQ9)

The scale was created by Kroenke et al. in 2001 and used to evaluate a person's depressed symptoms [40]. The scale had 9 items and rates each one using a 4-grade method. The total scores ranged from 0 to 27, while the scores for each item ranged from 0 (not at all) to 3 (nearly every day). The severity of depressive symptoms can be classified into the following phases based on the total score: "0–4" denotes the absence of depressive symptoms, "5–9" shows the presence of depressive symptoms, "10–14" denotes the presence of major depressive symptoms, and "15" and above denotes severe depressive symptoms. The Cronbach's alpha coefficient for this scale was 0878 and the KMO measure was 0.853.

### Quality of life scale(SF-12)

The scale was a 12-entry scale extracted from the SF-36 scale developed by the U.S. Bureau of Medical Research of the BoS in 1996 [41]. The present study utilized this scale to evaluate the quality of life of middle-aged and older adults hospitalized with chronic diseases. The scale had 8 dimensions: Physical Functioning (PF), Role-Physical (RP), Bodily Pain (BP), General Health (GH), Vitality (VT), Social Functioning (SF), Role-Emotional (RE), and Mental Health (MH). Greater scores indicate a greater level of life; the range is 0 to 100. The Cronbach's alpha coefficient for this scale was 0.862 and the KMO measure was 0.836.

### Covariates

Age, gender, residence status, health insurance status, physical activity, social activity, chronic pain, and health level, were included in our study as covariates. Age was categorized into three groups: 45–60 years, 61–74 years, and ≥ 75 years. Gender was classified into two groups: male and female. Residence status was segmented into four types: living with a spouse, living with other relatives, living alone, and other living arrangements. Health insurance status was divided into four categories: public medical insurance, private medical insurance, other insurance types, and none. For physical activity, we recognized three levels: Regular activity: Aerobic exercises performed 3–5 times per week for at least 30 min per session, at an intensity recommended by a healthcare professional. Irregular activity: Physical activity occurring less than 3 times per week or lasting less than 30 min per session, without a structured plan. Inactivity: not doing any additional physical activity other than the basic actions of daily living, such as walking, getting up, etc. Social interaction was categorized into three: No social activity: the patient does not participate in any organized, planned social activities. Occasional social activity: Patients partake in 1–2 organized social activities per month. Regular social activity: Patients attend at least one organized social activity weekly. Chronic pain was differentiated into: Yes: Persistent or recurring pain lasting more than three months. No: Absence of such pain. Finally, health level was parsed into five categories: Very poor: Patients experience severe health issues, resulting in significant functional impairment, necessitating consistent medical care. Poor: Frequent health challenges requiring routine medical attention. Fair: Stable health that still demands regular medical oversight. Good: Patients are mostly stable, with occasional medical interventions. Very good: Well-managed health conditions with minimal medical attention.

### Statistical analysis

Data were analyzed using IBM SPSS version 25.0 (IBM Corp, Armonk, NY). Measurement data were expressed as means and standard deviations, while count data were presented as frequencies and percentages. Pearson correlation analysis was employed to describe the relationship between variables. Regression analysis was used to investigate the association between psychological resilience and quality of life. To provide a detailed understanding of this relationship, four models were formulated. These models progressively controlled for potential confounding factors, giving clearer insight into the independent influence of each covariate on the predicted outcomes. Each model controlled for different sets of covariates, which helped clarify the magnitude and direction of the effects of the primary factors. The structural equation model was constructed using Amos 24.0, with the Bootstrap method applied to verify mediating effects. An alpha value of 0.05 was used for the test.

## Results

### Characteristics of participants

Our study comprised 339 participants. Table 1 presents the demographic characteristics of the study population and the univariate analyses of the quality of life for different characteristics. Out of the 339 hospitalized patients with chronic diseases, 207 (61.1%) were men and 132 (38.9%) were women. The patients' ages ranged from 45 to 96 years, with a mean age of 64.06 ± 10.01 years. The majority of the participants (59.9%) reported irregular physical activity, while 31.9% indicated experiencing chronic pain. Variations in the quality of life-based on residence status, health insurance status, and health levels are detailed in Table 1. Additionally, the distribution of chronic diseases across different organ systems is illustrated in Table 2.

**Table 1:** One-way analysis of the quality of life of patients hospitalized for chronic diseases with different characteristics (*N* = 339)

Variables	Group	N(%)	Mean ± SD	*F/t*	*P*
Age	45–60	133 (39.2%)	76.77 ± 10.36	0.861	0.773
	61–74	159 (46.9%)	74.71 ± 10.09		
	≥ 75	47 (13.7%)	73.65 ± 9.47		
Gender	Male	207 (61.1%)	75.09 ± 9.55	0.989	0.542
	Female	132 (38.9%)	75.79 ± 11.07		
Residence situation	and spouse	265 (78.2%)	75.62 ± 10.52	2.260	< 0.001
	With a relative other than your spouse	51 (15.0%)	74.56 ± 9.09		
	Living alone	18 (5.3%)	75.04 ± 7.81		
	Other	5 (1.5%)	75.37 ± 10.15		
Medical insurance situation	Publicly funded medical care	15 (4.4%)	74.03 ± 13.35	2.163	< 0.001
	Medical insurance	271 (79.9%)	75.64 ± 10.08		
	Other health insurance	31 (9.1%)	74.68 ± 9.47		
	None	14 (4.1%)	72.87 ± 9.77		
Physical Activity	Regular	88 (26.0%)	79.11 ± 9.47	1.064	0.410
	Irregular	203 (59.9%)	75.18 ± 9.68		
	Inactive	48 (14.2%)	69.23 ± 10.46		
Social Activity	Do not participate	63 (18.6%)	71.61 ± 9.79	0.928	0.655
	Occasional participation	207 (61.1%)	75.09 ± 10.01		
	Regularly participate	69 (20.4%)	79.65 ± 9.49		
Chronic pain	Yes	108 (31.9%)	71.63 ± 8.97	1.238	0.185
	No	231 (68.1%)	77.12 ± 10.23		
Health Level	Very poor	25 (7.4%)	67.76 ± 9.08	2.968	< 0.001
	Poor	117 (34.5%)	70.85 ± 8.61		
	Fair	161 (47.5%)	77.12 ± 8.51		
	Good	32 (9.4%)	86.88 ± 8.96		
	Very good	4 (1.2%)	92.41 ± 12.02

**Table 2:** The prevalence of chronic diseases in each organ system(*N* = 339)

Disease Distribution	Yes, N (%)
1. Heart	98 (29.0%)
2. Blood vessels	149 (44.0%)
3. Hematopoietic system (bone marrow)	40 (11.8%)
4. Respiratory	88 (26.0%)
5. Eye, ear, nose, and throat	36 (10.9%)
6. Upper gastrointestinal tract	50 (14.7%)
7. Lower gastrointestinal tract	62(18.3%)
8. Liver	107 (31.6%)
9. Kidney	51 (15.0%)
10. Urogenital	50 (14.7%)
11. Motor system	95 (28.0%)
12. Nervous system	89 (26.3%)
13. Endocrine/Metabolic and Breast Cancer	102 (30.1%)

### Correlation analysis of key variables

According to the Pearson correlation study, psychological resilience was positively related to quality of life and negatively related to depression and sleep quality (*p* < 0.01). Sleep quality exhibited a negative association with quality of life and a positive link with depression (*p* < 0.01); depression and quality of life had a negative correlation (*p* < 0.01). See Table 3.

**Table 3:** Descriptive statistics and correlation analysis of CD-RISC, PSQI, PHQ9, and SF-12

Variables	Mean ± SD	1	2	3	4
CD-RISC	49.65 ± 13.79	1			
PSQI	6.57 ± 3.39	-0.233^**^	1		
PHQ9	7.09 ± 4.40	-0.325^**^	0.398^**^	1	
SF-12	75.37 ± 10.16	0.389^**^	-0.384^**^	-0.494^**^	1

Note: ^∗∗^ represents *P* < 0.01

### Multiple linear regression analysis

Four replicate multiple linear regression analyses were conducted with quality of life as the dependent variable, sociodemographic characteristics as control variables, and psychological resilience, sleep status, and depression as the main independent variables (see Table 4). According to Model 1, physical activity, social activity, chronic pain, and health level had a significant impact on quality of life, with the general information variable explaining 31.3% of the criterion variables (*F* = 20.231, *ΔR*^*2*^ = 0.329, *P* < 0.001). Based on model 1, multiple linear regression analysis in Model 2 showed that psychological resilience was significantly positively associated with quality of life, explaining 37.0% of the standard variance (*F* = 23.054, *ΔR*^*2*^ = 0.058, *P* < 0.001). Model 3 added sleep quality on the base of model 2. Psychological resilience and quality of life were positively correlated, and sleep quality was significantly negatively correlated with quality of life, explaining 41.3% of the standard variance (*F* = 24.796, *ΔR*^*2*^ = 0.044, *P* < 0.001). Model 4 added depression to model 3, psychological resilience and quality of life were positively correlated, and sleep quality and depression were significantly negatively correlated with quality of life, explaining 46.3% of the standard variance (*F* = 27.450, *ΔR*^*2*^ = 0.050, *P* < 0.001). Consequently, sleep quality and depression act as mediators in the relationship between psychological resilience and quality of life. See Table 4.

**Table 4:** Multiple linear regression analysis results

Variables	Model 1				Model 2				Model 3				Model 4			
	S. E	B(95%CI)	t	p	S.E	B(95%CI)	t	p	S.E	B(95%CI)	t	p	S.E	B(95%CI)	*t*	*p*
Age	0.049	-0.060 (-0.156,0.037)	-1.220	0.223	0.047	-0.058 (-0.150,0.034)	-1.233	0.219	0.045	-0.047 (-0.136,0.042)	-1.050	0.295	0.043	-.0460 (-0.131,0.039)	-1.059	0.290
Gender	0.983	-0.041 (-1.974,1.893)	-0.042	0.967	0.941	0.066 (-1.785,1.918)	0.070	0.944	0.916	0.663 (-1.139,2.465)	0.724	0.470	0.877	0.726 (-0.999,2.451)	0.828	0.408
Residence status	0.743	0.239 (-1.222,1.700)	0.322	0.748	0.711	0.250 (-1.149,1.649)	0.352	0.725	0.687	0.336 (-1.014,1.697)	0.490	0.624	0.657	0.475 (-0.818,1.768)	0.722	0.471
Medical insurance status	0.909	0.176 (-1.613,1.965)	0.194	0.846	0.871	0.330 (-1.384,2.044)	0.379	0.705	0.841	0.476 (-1.179,2.131)	0.565	0.572	0.808	0.840 (-0.749,2.429)	1.040	0.299
Physical activity	0.789	-1.944 (-3.497, -0.392)	-2.463	0.014	0.764	-1.323 (-2.825,0.180)	-1.731	0.084	0.743	-0.877 (-2.338,0.584)	-1.181	0.239	0.717	-0.334 (-1.745,1.077)	-0.465	0.642
Social activity	0.086	2.019 (0.434,3.604)	2.505	0.013	0.773	1.738 (0.217,3.258)	2.247	0.025	0.746	1.815 (0.347,3.284)	2.432	0.016	0.719	2.299 (0.884,3.714)	3.196	0.002
Chronic pain	1.039	3.307 (1.264,5.350)	3.184	0.002	0.998	2.867 (0.904,4.829)	2.874	0.004	0.963	2.742 (0.848,4.637)	2.847	0.005	0.930	2.055 (0.226,3.884)	2.210	0.028
Health Level	0.620	5.383 (4.164,6.603)	8.686	< 0.001	0.600	4.905 (3.726,6.085)	8.181	< 0.001	0.586	4.449 (3.297,5.602)	7.595	< 0.001	0.568	3.943 (2.826,5.060)	6.944	< 0.001
Psychological resilience					0.033	0.185 (0.120,0.251)	5.563	< 0,001	0.033	0.158 (0.094,0.223)	4.862	< 0.001	0.032	0.121 (0.058,0.184)	3.795	< 0.001
PSQI									0.133	-0.667 (-0.929, -0.406)	-5.021	< 0.001	0.133	-0.444 (-0.706, -0.181)	-3.329	0.001
PHQ9													0.109	-.0608 (-0.822, -0.394)	-5.584	< 0.001
*F*		20.231				23.054				24.796				27.450		
*P*		< 0.001				< 0.001				< 0.001				< 0.001		
Adjusted *R*^*2*^		0.313				0.370				0.413				0.463		
*ΔR*^*2*^		0.329				0.058				0.044				0.050		

### Mediating effect analyses

**Fig. 2:**
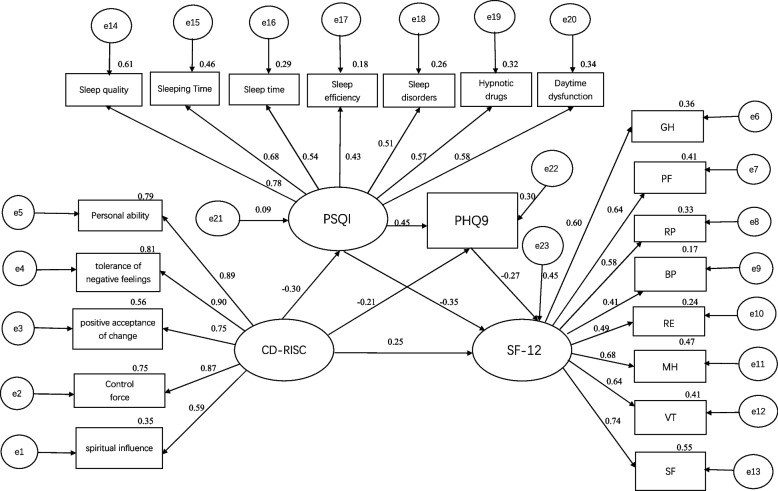
Chain mediation model between sleep quality, depression in psychological resilience, and quality of life

**Fig. 3:**
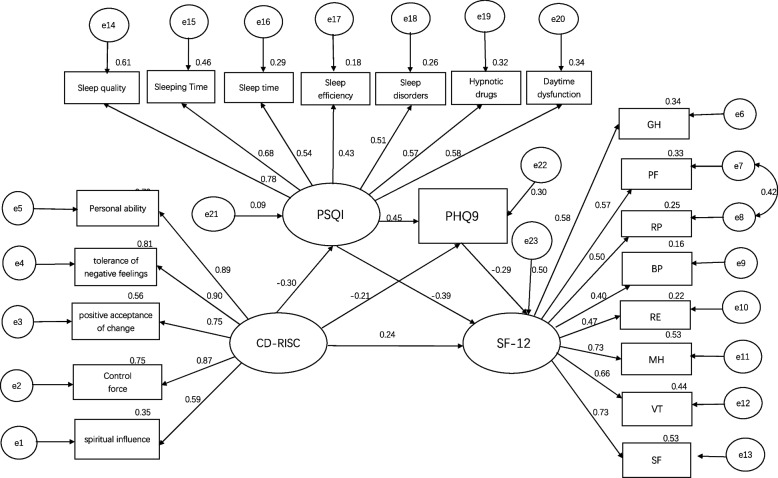
Modified chain mediator model between sleep quality, depression in psychological resilience, and quality of life

Amos 24.0 was used to construct a structural equation model with psychological resilience as the exogenous latent variable and sleep status, depression, and quality of life as the endogenous latent variables, and the structural model was estimated and tested using the great likelihood method. See Fig. 2. The model was corrected once according to the correction index, and the corrected model is shown in Fig. 3. Both the pre-correction and post-correction fit indices indicate that the model fits well were acceptable, and was better after correction, as shown in Table 5. The bias-corrected nonparametric percentile Bootstrap method was used to repeat the sampling 5000 times and set 95% confidence intervals for the mediating effect test, and the results of the mediating effect analysis of the modified model are shown in Table 6. The results showed that the direct effect of psychological resilience on quality of life was 0.239 which accounted for a total effect of 52.98%; There was a significant mediating effect of sleep quality and depression between psychological resilience and quality of life, the effect value was 0.213 and the total effect accounted for 47.02%. In this study, the total mediating effect consisted of indirect effects arising from three mediating pathways: Psychological resilience → sleep quality → quality of life (path 1) had a mediating effect of 0.115, which accounted for a total effect of 25.39%; Psychological resilience → depression → quality of life (path 2) had a mediating effect of 0.060, which accounted for a total effect of 13.24%; Psychological resilience → sleep quality → depression → quality of life (path 3) had a mediating effect of 0.038, which accounted for 8.39% of the total effect. The paths all reached a significant level as the model tests matched confidence intervals that did not contain 0, which is a sign of significance.

**Table5:** Applicability index of structural equation model

Adaptability Index	Preset Model	Correction Model	Criteria	Assessment
CMIN/DF	3.767	3.495	3–5	Acceptable
RMSEA	0.090	0.086	< 0.09	Acceptable
AGFI	0.788	0.806	> 0.8	Acceptable
GFI	0.831	0.846	> 0.8	Acceptable
NFI	0.786	0.803	> 0.8	Acceptable
CFI	0.832	0.849	> 0.8	Acceptable
IFI	0.833	0.851	> 0.8	Acceptable
TLI	0.808	0.827	> 0.8	Acceptable
PNFI	0.689	0.699	> 0.5	Acceptable
PGFI	0.662	0.670	> 0.5	Acceptable

**Table 6:** A test of mediating effects between psychological resilience and quality of life Note: X is Psychological resilience; M1 is Sleep quality; M2 is Depression; Y is Quality of life

Path	Effect Value	S.E	LLCI	ULCI	Effectiveness ratio %
Direct effect					
X → Y	0.239	0.059	0.125	0.354	52.98
Intermediary effect					
X → M1 → Y	0.115	0.037	0.056	0.201	25.39
X → M2 → Y	0.060	0.023	0.023	0.114	13.24
X → M1 → M2 → Y	0.038	0.013	0.019	0.074	8.39
Total intermediation effect	0.213	0.038	0.145	0.294	47.02
Total effect	0.453	0.056	0.340	0.555	100

## Discussion

Our study explored the association between psychological resilience and quality of life in middle-aged and older adults hospitalized with chronic diseases, and for the first time analyzed the role of sleep quality and depression in this process. The outcomes of the survey indicated a significant positive correlation between psychological resilience and quality of life in participants aged 45 years and older, and this correlation was partially mediated by sleep quality and depression.

From the results of this investigation, we found that the psychological resilience of middle-aged and older adults hospitalized with chronic diseases has a significant and direct impact on their quality of life, which agrees with the results of earlier studies [42, 43]. More specifically, middle-aged and older adults hospitalized with chronic diseases who have lower levels of psychological resilience tend to experience poorer quality of life [44]. The reason for this: On the one hand, hospitalized patients with low psychological resilience usually lack flexibility and adaptability, and are prone to negative emotions, complaints, and feelings of helplessness when facing unfamiliar healthcare environments and complex treatment processes, which in turn affects their quality of life [45]. On the other hand, hospitalized patients with lower levels of psychological resilience often lack the self-confidence to manage their illness effectively; they may doubt their abilities and control, leading to a reduced sense of self-efficacy, and this reduced self-efficacy may limit their ability to actively engage in self-management and actively seek appropriate support, thus leading to a further reduced quality of life [46]. Contrarily, middle-aged and older adults hospitalized with chronic diseases who exhibit higher levels of psychological resilience frequently have better quality of life [14]. This may be because hospitalized patients with higher psychological resilience are not only able to adapt quickly to new environments and cope with emergencies but also have excellent problem-solving skills, which enables them to feel the satisfaction of self-actualization in the face of adversity as well, thus obtaining a higher quality of life [47]. Furthermore, In addition, a high level of psychological resilience also enables hospitalized patients to accept reality more courageously, cooperate with treatment more positively, and maintain an optimistic attitude toward the future recovery process, which can significantly contribute to the promotion of disease recovery and the improvement of quality of life [48]. Therefore, future research should consider psychological resilience as an entry point for interventions aimed at improving the level of psychological resilience in middle-aged and older adults hospitalized with chronic diseases. Cultivating and improving the psychological resilience of hospitalized patients can help them better cope with the challenges of chronic diseases, thus improving their quality of life and well-being.

The findings revealed that psychological resilience can compromise the quality of life of middle-aged and older adults hospitalized with chronic diseases through the quality of sleep as a mediator. The psychological resilience level of Middle-aged and older adults hospitalized with chronic diseases, who undergo a variety of physical and psychological symptoms and the painful stress associated with diagnosis, treatment, and disease, is often lower than that of the general population [49]. Hospitalized patients with low levels of psychological resilience often show panic and helplessness in the face of disease stress and respond in a negative inactive way, which causes them to show more worry, fear, and insomnia, which can influence their sleep quality [50]. Weak sleep quality could seriously affect the physical function, daytime activity function, immune function, and cognitive ability of middle-aged and older adults hospitalized with chronic diseases, which in turn affects their quality of life [18, 51, 52]. Conversely, hospitalized patients with higher levels of psychological resilience can actively mobilize internal protective factors against disease stress in the face of stress brought about by various chronic diseases, such as relieving stress due to disease through distraction and positive psychological suggestions, thus improving sleep quality [53]. Good sleep quality helps to accelerate protein synthesis and tissue repair in the body, enhance the mental and physical strength of hospitalized patients, achieve physical and mental balance, and thus improve their quality of life [54]. Therefore, we should pay attention to the sleep of middle-aged and older adults hospitalized with chronic diseases and take timely and targeted actions to improve their sleep quality to strengthen their quality of life.

Our findings suggested that psychological resilience affects the quality of life of middle-aged and older adults hospitalized with chronic diseases through depression as a mediator. Middle-aged and older adults hospitalized with chronic diseases usually face more stress and survival challenges, which can have an impact on their psychological resilience [55]. Psychological resilience has been recognized as a protective factor for an individual's mental health [56]. Those patients with low levels of psychological resilience usually have difficulty coping positively and optimistically with various difficulties in treatment or life, such as pain, fatigue, and high hospitalization costs. This predicament may lead them to lose confidence in overcoming the disease and thus fall into negative emotional states such as anxiety and depression [31, 57]. Notably, negative emotions such as depression may also affect the quality of life of hospitalized patients [58]. On a physiological level, depression can adversely affect patients' cognitive functions, including memory, attention, cognitive sequencing ability, and information processing speed, which reduces the quality of life of hospitalized patients [59, 60]. On the psychological level, depression is mainly manifested as Demoralization Syndrome, including feelings of frustration, failure, emotional upset, meaninglessness, and helplessness, which can also hurt the quality of life of hospitalized patients [61]. Therefore, in addition to paying attention to the psychological resilience of middle-aged and older adults hospitalized with chronic diseases, healthcare professionals should also focus on patients with depressive tendencies to implement individualized interventions as early as feasible to enhance their quality of life.

Furthermore, the main finding of this study was that psychological resilience affects the quality of life of middle-aged and older adults hospitalized with chronic diseases through a sleep quality-depression chain mediating role. This result can be better explained by the Stress Process Theory [62]. In this theory, negative events suffered by middle-aged and older adults hospitalized with chronic diseases are stressors, psychological resilience can be considered as a cognitive evaluation factor of individuals, sleep disturbances and depressive symptoms when they occur are stress reactions, while the quality of life of hospitalized patients is considered as the outcome produced by stress reactions. Specifically, middle-aged and older adults hospitalized with chronic diseases often face negative events such as disease symptoms, decreased physical functioning, unsatisfactory treatment outcomes, communication barriers with healthcare professionals, and social role changes [63, 64]. These negative events as stressors may negatively affect patients' psychological state and mood, thus affecting their level of psychological resilience [65]. Hospitalized patients with lower levels of psychological resilience are usually accompanied by more physical and mental health problems, such as breathing difficulties, chronic pain, or other physical discomfort [66]. These health problems may lead to insomnia, frequent night awakenings, and other conditions that reduce sleep quality [67]. However, chronic poor sleep quality, insufficient sleep duration, and sleep disruption may trigger neurochemical imbalances, such as reduced brain levels of serotonin and dopamine, which in turn adversely affect mood regulation that contributes to the emergence or exacerbation of depression [68]. Depressive symptoms can negatively affect a hospitalized patient's mental health, daily functioning, interpersonal relationships, and physical health, thereby reducing their overall quality of life [69]. As a result, the results of this study support the stress process theory and emphasize the importance of psychological resilience, sleep quality, and depression on the quality of life of middle-aged and older adults hospitalized with chronic diseases. Overall, these findings remind us that in the management of middle-aged and older adults hospitalized with chronic diseases, it is important to emphasize the development of psychological resilience, pay attention to the improvement of sleep quality, and provide timely interventions for depression so that the general quality of life of the hospitalized patients can be improved.

In conclusion, this research aimed to comprehensively discover the potential factors of the relationship between psychological resilience and quality of life in middle-aged and older adults hospitalized with chronic diseases and to offer helpful messages for early identification and prevention of quality-of-life decline, which has certain theoretical and practical significance. Based on the above findings, we suggest that healthcare professionals should pay attention not only to hospitalized patients' psychological resilience but also to their sleep quality and depressive symptoms when improving the quality of life of middle-aged and older adults hospitalized with chronic diseases. The specific recommended measures are as follows: First, psychological resilience training programs, such as cognitive behavioral therapy and positive thought-based interventions, can be implemented to enhance the level of psychological resilience in hospitalized patients [70]. This will help them develop positive thinking patterns that improve their ability to cope with stress and negative emotions. Second, provide advice on sleep management, such as establishing good sleep habits, creating a comfortable sleep environment, and using relaxation techniques to improve the quality of hospitalized patients' sleep [71]. Gaining a good night's sleep is crucial to physical and mental health, which can boost a hospitalized patient's energy as well as their overall quality of life. Moreover, it is also important to provide recreational activities and social support for hospitalized patients with chronic diseases, for example, organizing activities such as cultural performances and social gatherings, which can reduce their loneliness, decrease the risk of depression, and increase the enjoyment of life [72, 73]. Through these comprehensive interventions, we hope to increase the psychological resilience of middle-aged and older adults hospitalized with chronic diseases, improve sleep quality, prevent depression, and ultimately improve their quality of life.

The main strengths of this study are as follows: first, this study was specifically designed for middle-aged and older adults hospitalized with chronic diseases, emphasizing the important value of increasing psychological resilience, improving sleep quality, and decreasing depression on the quality of life of this specific population, and providing a theoretical basis for clinicians and caregivers. Second, this study is the first to use a multiple mediator model to explore the relationship between psychological resilience, sleep quality, depression, and quality of life in middle-aged and older adults hospitalized with chronic diseases. Compared with the traditional single model, this approach allowed us to consider the interactions between these factors more comprehensively.

The study contained a few limitations. First of all, as this study was cross-sectional and was unable to establish a causal relationship between psychological resilience and quality of life, future longitudinal studies should address this problem and thoroughly examine the underlying mechanisms. Second, although this study draws some conclusions regarding psychosocial resilience, sleep quality, depression, and quality of life in middle-aged and older adults hospitalized with chronic diseases, it is important to note that these factors may vary in different settings. For example, in hospitals and in the community different environmental factors, social interactions, and the stresses and challenges of daily life may have different impacts on the above factors. Therefore, more research and validation are needed before applying these findings directly to the community or other settings.

## Conclusions

The objective of this research was to examine the mediating role of sleep quality and depression in the relationship between psychological resilience and quality of life in middle-aged and older adults hospitalized with chronic diseases. The research results revealed that psychological resilience had a significant effect on the quality of life, part of which was achieved through two mediators, sleep quality and depression, with an overall mediating effect of 47.02%. Given the generally low quality of life situation of middle-aged and older adults hospitalized with chronic diseases, it is especially important to take multifaceted measures to improve their quality of life. Therefore, healthcare professionals and stakeholders should pay attention to the sleep status and mental health of middle-aged and older adults hospitalized with chronic diseases, enhance their attention to psychological resilience, as well as to provide interventions and treatment measures for middle-aged and older adults hospitalized with chronic diseases, who have sleep problems and depressive tendencies.

## Data Availability

The datasets used and analyzed during the current study are available from the corresponding author upon reasonable request.

## Abbreviations

PHQ9: Patient Health Questionnaire-9
SD: Standard Deviation
SE: Standard Error
B: Unstandardized
CI: Confidence interval
ANOVA: Analysis of Variance
*ΔR*: Amount of R2 change
RMSEA: Root Mean Square Error of Approximation
AGFI: Adjusted Goodness of Fit Index
GFI: Goodness of Fit Index
NFI: Normative Fit Index
CFI: Comparative fit index
IFI: Invaluable Adaptation Index
TLI: Taker-Lewis Index
PNFI: Parsimonious Normative Fit Index
PGFI: Parsimonious Goodness of Fit Index
CMIN: Cardinality
DF: Degrees of Freedom
